# Radiation Oncologists’ Perspectives on Oligometastatic Prostate Cancer: A Survey from Korean Oligometastasis Working Group

**DOI:** 10.3390/curroncol31060245

**Published:** 2024-06-03

**Authors:** Gyu Sang Yoo, Sunmin Park, Chai Hong Rim, Won Kyung Cho, Ah Ram Chang, Young Seok Kim, Yong Chan Ahn, Eui Kyu Chie, On behalf of the Oligometastasis Working Group, Korean Cancer Association

**Affiliations:** 1 Department of Radiation Oncology, Chungbuk National University Hospital, Cheongju 28644, Republic of Korea; 2 Department of Radiation Oncology, Ansan Hospital, Korea University Medical College, Seoul 15355, Republic of Korea; 3 Department of Radiation Oncology, Samsung Medical Center, Sungkyunkwan University School of Medicine, Seoul 06351, Republic of Korea; 4 Department of Radiation Oncology/Cyberknife Center, Soonchunhyang University Seoul Hospital, Soonchunhyang University College of Medicine, Seoul 04401, Republic of Korea; 5 Department of Radiation Oncology, Asan Medical Center, Ulsan University School of Medicine, Seoul 05505, Republic of Korea; 6 Department of Radiation Oncology, Seoul National University College of Medicine, Seoul 03080, Republic of Korea

**Keywords:** local therapy, oligometastasis, prostate cancer, radiation therapy, survey study

## Abstract

Background: Interest in the oligometastatic prostate cancer (OMPC) is increasing, and various clinical studies have reported the benefits of metastasis-directed radiation therapy (MDRT) in OMPC. However, the recognition regarding the adopted definitions, methodologies of assessment, and therapeutic approaches is diverse among radiation oncologists. This study aims to evaluate the level of agreement for issues in OMPC among radiation oncologists. Methods: We generated 15 key questions (KQs) for OMPC relevant to definition, diagnosis, local therapies, and endpoints. Additionally, three clinical scenarios representing synchronous metastatic prostate cancer (mPC) (case 1), metachronous mPC with visceral metastasis (case 2), and metachronous mPC with castration-resistance and history of polymetastasis (case 3) were developed. The 15 KQs were adapted according to each scenario and transformed into 23 questions with 6–9 per scenario. The survey was distributed to 80 radiation oncologists throughout the Republic of Korea. Answer options with 0.0–29.9%, 30–49.9%, 50–69.9%, 70–79.9%, 80–89.9%, and 90–100% agreements were considered as no, minimal, weak, moderate, strong, and near perfect agreement, respectively. Results: Forty-five candidates voluntarily participated in this study. Among 23 questions, near perfect (*n* = 4), strong (*n* = 3), or moderate (*n* = 2) agreements were shown in nine. For the case recognized as OMPC with agreements of 93% (case 1), near perfect agreements on the application of definitive radiation therapy (RT) for whole metastatic lesions were achieved. While ≥70% agreements regarding optimal dose-fractionation for metastasis-directed RT (MDRT) has not been achieved, stereotactic body RT (SBRT) is favored by clinicians with higher clinical volume. Conclusion: For the case recognized as OMPC, near perfect agreement for the application of definitive RT for whole metastatic lesions was reached. SBRT was more favored as a MDRT by clinicians with a higher clinical volume.

## 1. Introduction

Oligometastatic disease (OMD) is a disease state in which a limited number of metastatic lesions exist while the disseminated metastatic growth is not fully developed [1]. This concept implies the possibility of the curation of OMD by applying ablative metastasis-directed local therapies (MDLTs). Since suggesting this concept, a number of studies have reported the benefit of MDLTs in improving oncological outcomes [2]. However, there is no consistency in the views of OMD among studies, including the definition and strategies for diagnosis, local treatment, and surveillance. To establish the consensus, the European Society for Radiotherapy and Oncology (ESTRO) conducted a survey study and reached a consensus on the definition of OMD [3]. However, this consensus does not consider primary tumor-specific aspects.

Prostate cancer (PC) is the second most common cancer and the fifth leading cause of cancer-related deaths among men worldwide [4]. Among men diagnosed with PC, approximately 20% have metastatic disease [5]. Interest in oligometastatic PC (OMPC) is increasing, and various clinical studies have reported the benefits of MDLT in OMPC. However, adopted definitions, methodologies of assessment, and therapeutic approaches vary among studies. Therefore, standardization of these issues according to the specific characteristics of OMPC is necessary for future investigation and clinical practice. For this requirement, a Korean Oligometastsais Working Group (K-OWG), affiliated with the Korean Cancer Association, was organized by radiation oncologists in the Republic of Korea to investigate and standardize various issues for OMPC. Herein, we raised OMPC-specific clinical issues that require consensus and performed a survey study to evaluate the level of agreement.

## 2. Materials and Methods

**Figure 1:**
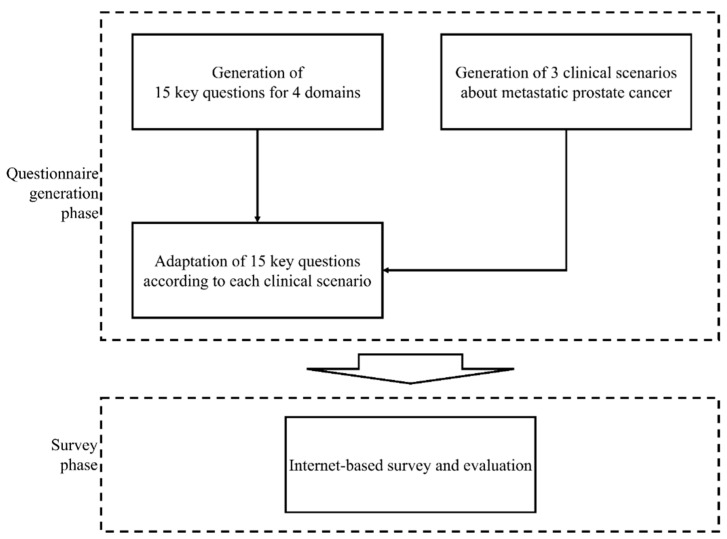
Flow of questionnaire generation and survey.

**Figure 2:**
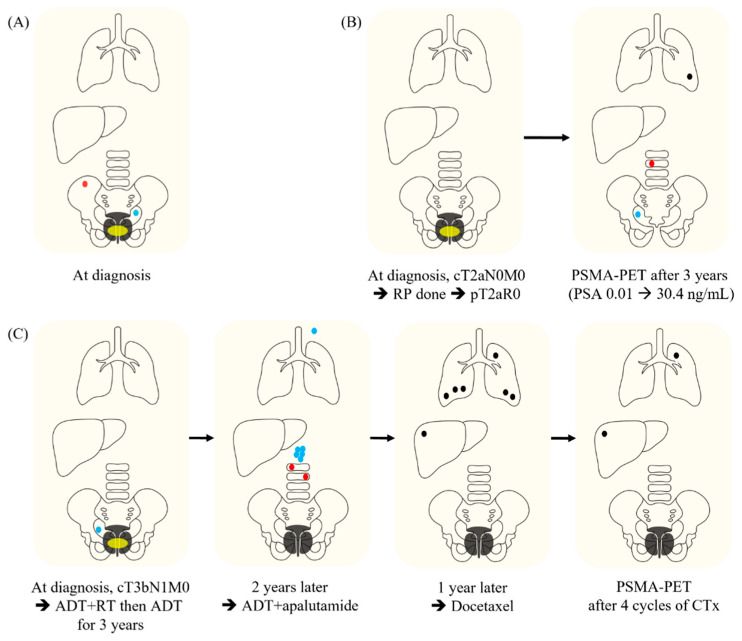
**Clinical scenarios for the survey.** (**A**) Case 1. A 74-year-old gentleman was shown to have a prostate-specific antigen (PSA) level of 24 ng/mL in a screening test. Transrectal ultrasonography (TRUS) and prostatic biopsy revealed a Gleason score of 8 (4 + 4), with adenocarcinoma in both sides. Magnetic resonance imaging (MRI) and computed tomography (CT) showed a prostatic mass with extraprostatic extension and right seminal vesicle invasion (yellow ellipse) and a lymph node (LN) with a short-axis size of 1 cm in the left obturator area (blue dot). An osteoblastic metastatic lesion with a long diameter of 2 cm in the right posterior iliac bone was confirmed on abdominopelvic (AP) CT and whole-body bone scan (WBBS). There is no evidence of other metastasis on CT and WBBS. Prostate-specific membrane antigen positron emission tomography (PSMA-PET) was not performed. (**B**) Case 2. A 65-year-old previously healthy gentleman was diagnosed with prostate cancer with clinical stage of T2aN0M0, a Gleason score of 7 (3 + 4), and a PSA of 12 ng/mL (yellow ellipse). The patient underwent a robotic radical prostatectomy. A pathological exam revealed the pathologic stage of pT2aN0 and clear resection margin. After surgery, the PSA level dropped to an undetectable level. However, PSA spiked to 30.4 ng/mL after 3 years. On PSMA-PET, metabolic uptakes were observed in the vertebral body of the 3rd lumber spine (red dot), right obturator LN (blue dot) and a nodule in the left lower lobe of the lung (black dot). There is no evidence of other metastasis. (**C**) Case 3. A 60-year-old previously healthy man was shown to have a PSA level of 13.8 ng/mL in a screening test. An initial workup revealed prostate adenocarcinoma in both sides of the prostate with a Gleason score of 9 (5 + 4) and clinical stage of T3bN1M0 (yellow ellipse). Two months after androgen-deprivation therapy (ADT), definitive radiation therapy of 70 Gy in 28 fractions was administered to the prostate, seminal vesicles, and elective nodal area. After 3 years of ADT administration, both prostate and right obturator lymph nodes showed complete remission, and PSA was maintained at 1.5 ng/mL without increase. However, the PSA increased to 2.1 ng/mL at 2 years after discontinuation of ADT and then continued to increase to 5.8 ng/mL 3 months later. AP CT and WBBS showed metastases in the 1st and 2nd lumbar spines (red dots), five or more para-aortic LNs (green dots), and left supraclavicular LN (brown dot). Therefore, ADT was resumed and apalutamide was administered. Thereafter, the PSA decreased to 1.5 ng/mL, and complete remission was confirmed by AP CT and WBBS. However, 1 year later, the PSA increased to 5 ng/mL again and 6 or more metastases were newly found in both lungs and liver with sizes of 2–3 cm on chest CT and AP CT (black dots). Bone metastasis was not noted. The patient was administered docetaxel every 3 weeks. AP CT and chest CT after four cycles of chemotherapy showed complete remission in metastatic tumors, except for metastatic lesions in the left upper lobe of the lung and segment 8 of the liver (black dots). In addition, the size of the persistent metastatic lesions increased from 1 cm to 1.5–2 cm. In PSMA-PET, no lesions showed metabolic uptake, other than the two persistent metastatic nodules. Abbreviations: RP, radical prostatectomy; PSMA-PET, prostate-specific membrane antigen positron emission tomography; PSA, prostate-specific antigen; ADT, androgen deprivation therapy; RT, radiation therapy; CTx, chemotherapy.

We raised five issues with OMPC including definition, diagnosis, radiation therapy (RT), and endpoint, and generated 15 relevant key questions (KQs) (Table 1). Additionally, three clinical scenarios regarding metastatic PC (mPC) were made for the survey. Case 1 is an mPC case with single pelvic lymph node (LN) and single right posterior iliac bone metastasis which were detected at the time of diagnosis for primary disease (synchronous mPC). Case 2 is an mPC case with a single lumbar spine, single pelvic LN, and single pulmonary metastasis at 3 years after radical prostatectomy for localized PC (de novo metachronous mPC). And Case 3 is a metastatic castration-resistant PC (CRPC) case with two lesions (pulmonary and hepatic metastases) showing induced oligoprogression according to the disease classification by ESTRO following definitive RT to the whole pelvis and androgen deprivation therapy (ADT) for localized high-risk PC and subsequent conversion to polymetastatic CRPC with six or more metastatic lesions [6]. The 15 KQs were adapted in the context of each clinical scenario and transformed into 23 multiple-choice questions with 6–9 per scenario. A questionnaire regarding the demographics of respondents was also developed with eight questions. The flow of the survey design is illustrated in Figure 1. Details of the scenarios are shown in Figure 2.

An anonymous survey was conducted using an online platform provided by SurveyMonkey^®^ (Palo Alto, CA, USA) in August 2022. The case scenarios and corresponding questionnaires were distributed to 80 certified radiation oncologists in the Republic of Korea who are members of the Genitourinary Division of the Korean Society of Radiation Oncology. The levels of agreement were defined as follows: answer options with 0.0–29.9%, 30–49.9%, 50–69.9%, 70–79.9%, 80–89.9%, and ≥90% agreements were considered as no, minimal, weak, moderate, strong, and near perfect agreement, respectively. Response rates were compared according to the average monthly numbers of new and OMD patients per year treated by responders using Fisher’s exact test.

## 3. Results

Of the 80 candidates, 45 (56.3%) voluntarily participated in the survey. Table 1 summarizes the demographic characteristics of responders. The results of the survey are shown in Supplementary Tables S1–S3. The characteristics of the cases and rates of response to the KQs according to the cases are presented in Table 1.

### 3.1. Definitions

There was a near perfect agreement in the response that the case was included in OMPC (93.0%) only for Case 1. All the responders who disagreed that Case 3 was included in the OMPC category replied that the previous history of polymetastatic disease could be the reason for excluding Case 3 in the OMPC category while only 28.6% selected CRPC status as the reason.

### 3.2. Diagnosis

For Case 1, only 60.5% agreed with the request of PSMA-PET to confirm the OMPC status. For Case 2, only 30.2% replied that the steep elevation of the prostate-specific antigen (PSA) could be the reason for excluding the case in the OMPC category.

### 3.3. Treatment

In Case 1, 93.0% of the responders replied that local treatment for primary PC was required and all chose definitive RT as the modality for primary PC. In Case 1, 97.7% of responders agreed with the application of MDLT, and all chose metastasis-directed RT (MDRT) for all metastatic lesions as MDLT while there was no moderate or higher level of agreement regarding the questions in Cases 2 and 3. To determine whether systemic therapy was required even if local control was achieved after MDRT, 79.1% and 87.2% responded that systemic therapy was required for Cases 1 and 3, respectively. No moderate or higher level of agreement has been reached regarding the dose-fractionation regimens for MDRT. However, there were significant differences in the response rates according to the clinical volume of new patients or OM patients treated by responders. For Case 2, the palliative RT dose to bone metastasis was chosen by responders with an average number of new patients per month < 30, significantly more than others (37.5% vs. 5.3%, *p =* 0.026; Supplementary Table S2). On the other hand, stereotactic body RT (SBRT) for lung metastasis was significantly preferred in responders with a clinical volume of the average numbers of OMD per year ≥ 10 (81.0% vs. 45.5%, *p* = 0.027; Supplementary Table S2). For Case 3, metastasis-directed SBRT was also significantly preferred in responders with a clinical volume of the average numbers of OMD per year ≥ 10 than others (80.0% vs. 42.1%, *p* = 0.022; Supplementary Table S3).

### 3.4. Endpoint

For the question about the parameter representing the failure of MDRT, responses in radiology and PSA level were chosen at 76.7% and 86.1%, respectively, for Case 1 while the rates of agreements were 81.4% and 72.1%, respectively, for Case 2 (Table 1). The rate of response that PSMA-PET needs to be applied to evaluate the complete remission after MDRT was 61.5% for Case 3.

## 4. Discussion

### 4.1. Definitions

In our study, only Case 1 was recognized as OMPC with a near perfect agreement. The definitions adopted by previous studies are heterogeneous in terms of number, location, and imaging modality for diagnosing OMPC. To provide a consensus, the ESTRO Guidelines Committee proposed consensus recommendations [7]. In the recommendation, 80% of the experts agreed to recommend MDRT for a maximum of five lesions [7]. In addition, 88% agreement was achieved in treating OMPC patients with LN, bone, and visceral metastasis. MDRT was not contraindicated by panelists; however, it was limited to selected cases based on clinical judgment [7].

While the majority of previous studies targeted hormone-sensitive mPC [8], oligometastatic CRPC has been investigated in only a limited number of studies [9,10]. The ESTRO recommendation also dealt with oligoprogressive CRPC without visceral metastasis [7]. In the Dutch multidisciplinary consensus meeting, 90% of the participants disagreed with the statement that OMPC following failure to ADT should preferably be treated by radical MDLT [11]. However, in our study, only 28.6% agreed that CRPC status was the reason for excluding Case 3 from the OMPC category. Therefore, this issue remains unclear. Rather than CRPC, a history of polymetastasis was recognized as the most important reason for excluding Case 3 from the OMPC category in our study. ESTRO and EORTC have suggested the concept of induced OMD with which patients have a history of polymetastasis before the diagnosis of OMD [6]. While previous studies have reported de novo OMD most frequently, induced OMD has rarely been reported [12]. The literature has not reported on induced OMPC, which might be a reason for the low awareness. Further studies are required to validate the concept of induced OMD in mPC.

### 4.2. Diagnosis

For the diagnosis of OMPC, the high performance of PSMA-PET has been proven in detecting metastasis of both hormone-sensitive PC and CRPC [13,14]. Based on this result, a consensus on the requirement of PSMA-PET for the confirmation of OMPC has been reached in previous studies [7,11]. However, in our study, there was no moderate or higher level of agreement regarding the requirement of PSMA-PET for Case 1 in which only conventional imaging was performed. Despite the excellent performance of PSMA-PET, its use was limited in the Republic of Korea because of limitations in government insurance coverage and this may impact the results of this study. However, the coverage was recently extended to include PSMA-PET, and the requirement for PSMA-PET is expected to increase in the Republic of Korea [15]. Therefore, re-evaluation of the consensus regarding PSMA-PET is necessary for the future.

There are no suggested criteria for the definition of OMPC, including the level or kinetics of the PSA [7]. In a Dutch multidisciplinary consensus meeting, more than 80% of panelists responded that PSA kinetics were not important for treatment decisions in synchronous OMPC, whereas more than 60% agreed that it was very important in metachronous OMPC [11]. In addition to PSA, several biomarkers including genetic markers, circulating tumor cells, and immunologic markers have been investigated showing impractical performance for predicting outcomes [16].

### 4.3. Treatment

In our study, near perfect agreements were achieved for the requirement of local treatment for primary tumors, MDRT for all metastatic lesions, and systemic treatment even if MDRT achieved local control of metastatic lesions in Case 1 which was the only one showing also near perfect agreement in the recognition as OMPC.

Based on previous trials demonstrating the benefits of RT for primary tumors, local RT for primary tumors for low-volume mPC is recommended in the guidelines [17,18]. The strong agreement on this issue for Case 1 may reflect the current recommendations. For metastatic lesions, protocols from relevant trials are commonly required to encompass whole lesions in the MDRT fields [19,20]. Our results showed a consistent agreement in Case 1. Despite no moderate or higher level of agreement that Cases 2 and 3 were included in the OMPC category, in the subgroups recognizing Cases 2 and 3 as OMPC, 86.7% and 94.4% agreed with the MDL(R)T for whole metastatic lesions, respectively. PSMA-PET can be utilized to select the RT target as emphasized in the ORIOLE trial [19].

Regarding MDRT for pelvic LN metastasis, whether to perform elective nodal RT (ENRT) or not also remains controversial [7]. In our study, the rate of agreement for ENRT for pelvic LN recurrence in Case 2 was only 23.3% (Supplementary Table S2). Previous studies have reported controversial results for the benefit of ENRT compared with local RT for pelvic LN recurrence from PC [21,22]. To address this issue, the PEACE V-STORM trial is ongoing [23]. In recommendations for RT in OMPC from an ESTRO consensus, ENRT with a boost on suspicious LNs was recommended as a preferred option but with only 60% agreement with divergent criteria for ENRT [7].

There was no moderate or higher level of agreement regarding radiation dose-fractionation schemes. In previous trials, the dose-fractionation scheme was heterogeneous among trials and anatomic sites [20]. In the ESTRO consensus recommendations, the preferred dose-fractionation regimens of SBRT for bone and LN were diverse; the results were 27–33 Gy in three fractions and 35–40 Gy in five fractions of which 30 Gy in three fractions and 35 Gy in five fractions were mostly preferred [7]. In our study, for responders with higher clinical volume for new patients or OMD patients, SBRT as MDRT was significantly favored. And RT with a palliative dose was significantly less preferred, implying that SBRT might be extended for OPMC as clinical experience is accumulated. Another controversial issue is the calculation of effective SBRT doses. Generally, SBRT with a biological effective dose (BED) of ≥100 Gy is recommended to improve local control [24]. To calculate BED, the radiation sensitivity of the primary tumor, which is reflected by α/β, should be considered [25,26]. PC has been recognized to have an α/β of 1.5–3.0 Gy, which is lower than that of other primary cancers, frequently with an α/β of 10 [27]. A previous study identified a BED greater than 100 Gy, calculated with an α/β ratio of 3 as a significant factor for local progression-free survival [26]. However, in the present study, an α/β ratio of 10 rather than 3 was preferred in the BED calculation to determine the SBRT dose for lung or liver metastasis. Similarly, a previous study argued that a higher α/β ratio should be used as the dose per fraction increases [28]. Therefore, this problem remains unsolved. Further studies are needed to determine the optimal dose of MDRT for OMPC.

### 4.4. Endpoint

In our study, moderate and strong agreements that the radiological response of the RT target could represent the outcome of MDRT were reached in Cases 1 and 2, respectively. For the PSA response, a strong agreement was achieved only in Case 1, and a moderate agreement was reached in Case 2. The emergence of new lesions was also considered as a parameter for the outcome of MDRT, with moderate agreement in cases 1 and 2. Because PSA is a specific marker for disease progression in PC, the response and kinetics of PSA after MDRT can be an important endpoint [7,29]. However, in the ESTRO consensus, the achievement of consensus failed regarding the definition of biochemical failure after MDRT for OMPC [7]. As the PSA can be a surrogate for disease progression even at the microscopic scale in PC, methodologies for the optimal use of the PSA after MDRT need to be established [11]. Our study showed only weak agreement regarding the use of PSMA-PET in evaluating an MDRT response. The role of PET in MDRT has been evaluated in PET-guided MDRT, particularly in the selection of target lesions [13,14,19]. However, for the evaluation of the RT response, the relevant role of PSMA-PET or choline-PET has been investigated in very few studies that showed a decrease in uptake values after RT [30]. This is also an important field of future research to build criteria for the treatment response evaluation of OMPC.

### 4.5. Limitations

This study had several limitations. This study does not represent the opinions of multidisciplinary experts, but only those of radiation oncologists in the Republic of Korea. Because the opinion of radiation oncologists could be influenced in the context of multidisciplinary discussion according to the cases, the cautious interpretation of the results in this study is required. The number of respondents was small and, especially, the rate of response was only 56.3%. This might not be enough to generalize the opinions of the radiation oncologists in the Republic of Korea. There was a limitation in increasing the rates of response because the survey should be based on the voluntary participation of responders. As the questionnaires were bound in the example cases, the agreements can be inconsistent for other possible clinical scenarios. The subsequent studies with more sufficient respondents and sample cases would be necessary to represent the opinion of radiation oncologists in the Republic of Korea and the diverse clinical situation regarding OMPC.

## 5. Conclusions

For the case recognized as OMPC, near perfect agreements were reached for the application of local therapies for primary and whole metastatic lesions. However, systemic therapy was also agreed to be applied very strongly even if local control was achieved after MDRT. While moderate or higher levels of agreement regarding the optimal dose-fractionation scheme for MDRT were not achieved, SBRT was favored by clinicians with a higher clinical volume for new patients or OMD patients. Further clinical studies on topics with an unreached consensus are necessary.

## Figures and Tables

**Table 1:** Questions and responses.

Questions	Response	Number
Questions for demographics of respondents		n (%)
Q1. How many years have you practiced as a radiation oncologist?	<10	15 (33.3)
	10–20	12 (26.7)
	20–30	15 (33.3)
	>30	3 (6.7)
Q2. What is the average number of new patient(s) treated by responder per month?	<10	4 (8.9)
	10–20	10 (22.2)
	20–30	11 (24.4)
	>30	20 (44.4)
Q3. Do you participate in the treatment of OMD?	Yes	44 (97.8)
	No	1 (2.2)
Q4. What is the average number of OMD patient(s) treated by responder per year?	<10	24 (53.3)
	10–20	6 (13.3)
	20–30	5 (11.1)
	>30	10 (22.2)
Q5. Which site do you apply SBRT to in your clinical practice? (multiple choices)	None	9 (20.0)
	Bone	30 (66.7)
	Lung	29 (64.4)
	Liver	20 (44.4)
	LN	17 (37.8)
	Brain	21 (46.7)
Key questions for clinical issues	Adapted questions and choices	n/total n (%)
*Definition* KQ1. Does the definition of OMPC depend on the sites of metastasis? KQ2. Are there criteria in the number of metastases or metastatic organs for the definition of OMPC? KQ3. Is the definition of OMPC limited to the concept of low-volume metastasis? KQ4. Are there criteria for the maximum size of metastases for the definition of OMPC? KQ5. Does the OMPC include the induced oligometastatic disease from polymetastatic disease after systemic therapy? KQ6. Is the concept of OMPC valid for CRPC?	*Which case can be included in OMPC? (multiple choices)*	
	Case 1 Case 2 Case 3	40/43 (93.0) 30/43 (69.8) 18/39 (46.2)
	*If Case 3 is not included in the category of OMPC, what can be the reason? (multiple choices)*	
	History of polymetastasis Progression to CRPC Insufficiency in test for confirmation of OMPC This case is included in the category of OMPC.	21/39 (53.8) 6/39 (15.4) 1/39 (2.6) 18/39 (46.2)
*Diagnosis*		
KQ7. Is there a PSMA-PET requirement to define OMPC?	*Is PSMA-PET required to confirm OMPC in Case 1?*	
	Yes No	26/43 (60.5) 17/43 (39.5)
KQ8. Is there any biochemical marker representing occult polymetastasis?	*Can a steep PSA increase be a marker for the exclusion of OMPC in Case 2?*	
	Yes No	13/43 (30.2) 30/43 (69.8)
*Treatment*		
KQ9. Should primary prostate cancer be controlled?	*Which local treatment do you recommend for primary tumor in Case 1? (multiple choice)*	
	Definitive treatment is not recommended. RP + PLND RP + PLND + adjuvant RT Definitive RT	3/43 (7.0) 0/43 (0.0) 6/43 (14.0) 40/43 (93.0)
KQ10. Are additional metastasis-directed local therapies including surgery, radiofrequency ablation, and/or RT for OMPC beneficial over the systemic therapy only? KQ11. Should metastasis-directed RT for OMPC encompass the whole metastatic disease burden?	*Is MDL(R)T required?*	
	For Case 1	
	Yes (MDRT for all metastatic lesions is required.)	42/43 (97.7)
	For Case 2 (multiple choices)	
	MDRT for all metastatic lesion Resection of lung metastasis and RT for other lesions	25/43 (58.1) 19/43 (44.2)
	For Case 3	
	RT for whole metastatic lesions	28/39 (71.8)
KQ12. Is cessation of systemic therapy possible if metastasis-directed RT alone shows the local control of oligometastases?	*Is STx required even if LC is achieved after MDRT?*	
	For Case 1 (multiple choices)	
	Yes	34/43 (79.1)
	ADT but if local treatment is applied and PSA levels are undetectable, discontinuation of ADT	12/43 (27.9)
	Systemic therapy is not recommended.	1/43 (2.3)
	For Case 2: NA	
	For Case 3	
	Yes No	34/39 (87.2) 5/39 (12.8)
KQ13. Is there a recommended optimal timing for the metastasis-directed RT for OMPC?	*What is the optimal timing for MDRT? (multiple choices)* For Case 1	
	Same timing as local treatment for primary tumor	40/43 (93.0)
	For Case 2	
	Concurrently with STx Prior to STx 2–3 months after initiation of STx When lesion-related symptoms develop while maintaining STx When PSA rises during the maintenance of systemic therapy	17/43 (39.5) 1/43 (2.3) 22/43 (51.2) 11/43 (25.6) 11/43 (25.6)
	For Case 3: NA	
KQ14. Are there recommended dose-fractionation regimens for the metastasis-directed RT for OMPC?	*What are the recommended dose-fractionation regimens for MDRT? (multiple choices)*	
	For Case 1	
	For pelvic bone metastasis	
	Palliative RT dose (eg., 30 Gy/10–12 fx, 20 Gy/4–5 fx, 8 Gy/1 fx, etc., or similar dose-fx scheme)	7/43 (16.3)
	SBRT (≤5 fractions) (eg., 18–24 Gy/1 fx, 20–24 Gy/2 fx, 21–30 Gy/3 fx, etc., or similar dose-fx scheme)	25/43 (58.1)
	Moderate dose RT (eg., 30–36 Gy/6 fx, 40–48 Gy/8 fx, 40–50 Gy/10 fx, 39–45 Gy/13–15 fx, etc., or similar dose-fx scheme)	18/43 (41.9)
	Definitive dose with conventional fx (eg., 50–60 Gy in 20–30 fx, etc., with 1.8–2.0 Gy per fx)	15/43 (34.9)
	For Case 2	
	For spine metastasis	
	Palliative RT dose SBRT (≤5 fractions) Moderate dose RT Definitive dose with conventional fx	10/43 (23.3) 30/43 (69.8) 13/43 (30.2) 2/43 (4.7)
	For LN metastasis	
	Palliative dose RT to LN only Hypofractionated high-dose RT to LN only High-dose conventional RT to LN only: 14.0 High-dose conventional RT to whole pelvis	3/43 (7.0) 29/43 (67.4) 6/43 (14.0) 10/43 (23.3)
	For lung metastasis	
	Palliative dose RT SBRT with BED_10_ ≥ 100 Gy SBRT with BED_3_ ≥ 100 Gy Moderate-dose RT Definitive dose with conventional fx	4/43 (9.3) 27/43 (62.8) 14/43 (32.6) 15/43 (43.9) 3/43 (7.0)
	For Case 3	
	For lung and liver metastasis	
	Palliative dose RT SBRT with BED_10_ ≥ 100 Gy SBRT with BED_3_ ≥ 100 Gy Moderate-dose RT Definitive dose with conventional fx	7/39 (17.9) 24/39 (61.5) 9/39 (23.1) 14/39 (35.9) 1/39 (2.6)
*Endpoint*		
KQ15. What endpoints are important for OMPC?	*Which parameter represents the outcome of MDRT? (multiple choices)*	
	For Case 1	
	Radiological response of RT target New lesion PSA response	33/43 (76.7) 32/43 (74.4) 37/43 (86.1)
	For Case 2	
	Radiological response of RT target New lesion PSA response	35/43 (81.4) 31/43 (72.1) 31/43 (72.1)
	For Case 3: NA *Do you think PSMA-PET is necessary to evaluate remission after RT in Case 3?*	
	Yes No	24/39 (61.5) 15/39 (38.5)

*n.* number, *OMD* oligometastatic prostate cancer, *SBRT* stereotactic body RT, *LN* lymph node, *KQ* key question, *OMPC* oligometastatic prostatic cancer, *CRPC* castration-resistant prostate cancer, *PSMA-PET* prostate-specific membrane antigen positron emission tomography, *PSA* prostate-specific antigen, *RP* radical prostatectomy, *PLND* pelvic lymph node dissection, *RT* radiation therapy, *NA* not applicable, *MDRT* metastasis-directed radiation therapy, *STx* systemic therapy, *LC* local control, *ADT* androgen deprivation therapy, *fx* fraction, *BED_x_* biologically effective dose with α/β = x.

## Data Availability

The data presented in this study are available upon request from the corresponding author.

## Supplementary Materials

The following supporting information can be downloaded at https://www.mdpi.com/article/10.3390/curroncol31060245/s1, Table S1: Questions and corresponding responses for Case 1; Table S2: Questions and corresponding responses for Case 2; Table S3: Questions and corresponding responses for Case 3.

## List of Abbreviations

ADT	Androgen deprivation therapy
BED	Biological effective dose
CRPC	Castration-resistant prostate cancer
ENRT	Elective nodal radiation therapy
ESTRO	European Society for Radiotherapy and Oncology
KQ	Key questions
LN	Lymph node
MDLT	Metastasis-directed local therapies
mPC	Metastatic prostate cancer
MDRT	Metastasis-directed RT
OMD	Oligometastatic disease
OMPC	Oligometastatic prostate cancer
PC	Prostate cancer
PSA	Prostate-specific antigen
PSMA-PET	Prostate-specific membrane antigen positron-emission tomography
RT	Radiation therapy
SBRT	Stereotactic body radiation therapy

## References

[1] Hellman S., Weichselbaum R.R. (1995). Oligometastases. J. Clin. Oncol..

[2] Rim C.H., Cho W.K., Lee J.H., Kim Y.S., Suh Y.G., Kim K.H., Chie E.K., Ahn Y.C., Oligometastasis Working Group K.C.A. (2022). Role of Local Treatment for Oligometastasis: A Comparability-Based Meta-Analysis. Cancer Res. Treat..

[3] Lievens Y., Guckenberger M., Gomez D., Hoyer M., Iyengar P., Kindts I., Mendez Romero A., Nevens D., Palma D., Park C. (2020). Defining oligometastatic disease from a radiation oncology perspective: An ESTRO-ASTRO consensus document. Radiother. Oncol..

[4] Sung H., Ferlay J., Siegel R.L., Laversanne M., Soerjomataram I., Jemal A., Bray F. (2021). Global Cancer Statistics 2020: GLOBOCAN Estimates of Incidence and Mortality Worldwide for 36 Cancers in 185 Countries. CA Cancer J. Clin..

[5] Rao A., Vapiwala N., Schaeffer E.M., Ryan C.J. (2019). Oligometastatic Prostate Cancer: A Shrinking Subset or an Opportunity for Cure?. Am. Soc. Clin. Oncol. Educ. Book.

[6] Guckenberger M., Lievens Y., Bouma A.B., Collette L., Dekker A., deSouza N.M., Dingemans A.C., Fournier B., Hurkmans C., Lecouvet F.E. (2020). Characterisation and classification of oligometastatic disease: A European Society for Radiotherapy and Oncology and European Organisation for Research and Treatment of Cancer consensus recommendation. Lancet Oncol..

[7] Zilli T., Achard V., Dal Pra A., Schmidt-Hegemann N., Jereczek-Fossa B.A., Lancia A., Ingrosso G., Alongi F., Aluwini S., Arcangeli S. (2022). Recommendations for radiation therapy in oligometastatic prostate cancer: An ESTRO-ACROP Delphi consensus. Radiother. Oncol..

[8] Connor M.J., Smith A., Miah S., Shah T.T., Winkler M., Khoo V., Ahmed H.U. (2020). Targeting Oligometastasis with Stereotactic Ablative Radiation Therapy or Surgery in Metastatic Hormone-sensitive Prostate Cancer: A Systematic Review of Prospective Clinical Trials. Eur. Urol. Oncol..

[9] Lohaus F., Zophel K., Lock S., Wirth M., Kotzerke J., Krause M., Baumann M., Troost E.G.C., Holscher T. (2019). Can Local Ablative Radiotherapy Revert Castration-resistant Prostate Cancer to an Earlier Stage of Disease?. Eur. Urol..

[10] Yoshida S., Takahara T., Arita Y., Ishii C., Uchida Y., Nakagawa K., Toda K., Sakamoto T., Kijima T., Yokoyama M. (2019). Progressive Site-Directed Therapy for Castration-Resistant Prostate Cancer: Localization of the Progressive Site as a Prognostic Factor. Int. J. Radiat. Oncol. Biol. Phys..

[11] Aluwini S.S., Mehra N., Lolkema M.P., Oprea-Lager D.E., Yakar D., Stoevelaar H., van der Poel H., Dutch Oligometastatic Prostate Cancer Working G., Busstra M., de Jong I.J. (2020). Oligometastatic Prostate Cancer: Results of a Dutch Multidisciplinary Consensus Meeting. Eur. Urol. Oncol..

[12] Nevens D., Jongen A., Kindts I., Billiet C., Deseyne P., Joye I., Lievens Y., Guckenberger M. (2022). Completeness of Reporting Oligometastatic Disease Characteristics in the Literature and Influence on Oligometastatic Disease Classification Using the ESTRO/EORTC Nomenclature. Int. J. Radiat. Oncol. Biol. Phys..

[13] Barbato F., Fendler W.P., Rauscher I., Herrmann K., Wetter A., Ferdinandus J., Seifert R., Nader M., Rahbar K., Hadaschik B. (2021). PSMA-PET for the assessment of metastatic hormone-sensitive prostate cancer volume of disease. J. Nucl. Med..

[14] Fendler W.P., Weber M., Iravani A., Hofman M.S., Calais J., Czernin J., Ilhan H., Saad F., Small E.J., Smith M.R. (2019). Prostate-Specific Membrane Antigen Ligand Positron Emission Tomography in Men with Nonmetastatic Castration-Resistant Prostate Cancer. Clin. Cancer Res..

[15] Hong J.H. (2022). An Update of Prostate-Specific Membrane Antigen Theranostics in Prostate Cancer. Korean J. Urol. Oncol..

[16] Surcel C., Kretschmer A., Mirvald C., Sinescu I., Heidegger I., Tsaur I. (2022). Molecular Mechanisms Related with Oligometastatic Prostate Cancer-Is It Just a Matter of Numbers?. Cancers.

[17] Burdett S., Boeve L.M., Ingleby F.C., Fisher D.J., Rydzewska L.H., Vale C.L., van Andel G., Clarke N.W., Hulshof M.C., James N.D. (2019). Prostate Radiotherapy for Metastatic Hormone-sensitive Prostate Cancer: A STOPCAP Systematic Review and Meta-analysis. Eur. Urol..

[18] National Comprehensive CAncer Network Prostate Cancer (Version 1.2023). https://www.nccn.org/professionals/physician_gls/pdf/rectal.pdf.

[19] Phillips R., Shi W.Y., Deek M., Radwan N., Lim S.J., Antonarakis E.S., Rowe S.P., Ross A.E., Gorin M.A., Deville C. (2020). Outcomes of Observation vs Stereotactic Ablative Radiation for Oligometastatic Prostate Cancer: The ORIOLE Phase 2 Randomized Clinical Trial. JAMA Oncol..

[20] Olson R., Mathews L., Liu M., Schellenberg D., Mou B., Berrang T., Harrow S., Correa R.J.M., Bhat V., Pai H. (2020). Stereotactic ablative radiotherapy for the comprehensive treatment of 1-3 Oligometastatic tumors (SABR-COMET-3): Study protocol for a randomized phase III trial. BMC Cancer.

[21] De Bleser E., Jereczek-Fossa B.A., Pasquier D., Zilli T., Van As N., Siva S., Fodor A., Dirix P., Gomez-Iturriaga A., Trippa F. (2019). Metastasis-directed Therapy in Treating Nodal Oligorecurrent Prostate Cancer: A Multi-institutional Analysis Comparing the Outcome and Toxicity of Stereotactic Body Radiotherapy and Elective Nodal Radiotherapy. Eur. Urol..

[22] Ost P., Jereczek-Fossa B.A., Van As N., Zilli T., Tree A., Henderson D., Orecchia R., Casamassima F., Surgo A., Miralbell R. (2016). Pattern of Progression after Stereotactic Body Radiotherapy for Oligometastatic Prostate Cancer Nodal Recurrences. Clin. Oncol..

[23] De Bruycker A., Spiessens A., Dirix P., Koutsouvelis N., Semac I., Liefhooghe N., Gomez-Iturriaga A., Everaerts W., Otte F., Papachristofilou A. (2020). PEACE V—Salvage Treatment of OligoRecurrent nodal prostate cancer Metastases (STORM): A study protocol for a randomized controlled phase II trial. BMC Cancer.

[24] Park S., Urm S., Cho H. (2014). Analysis of biologically equivalent dose of stereotactic body radiotherapy for primary and metastatic lung tumors. Cancer Res. Treat..

[25] Sanchez-Iglesias A.L., Morillo-Macias V., Santafe-Jimenez A., Ferrer-Albiach C. (2022). Bone-only oligometastatic prostate cancer: Can SABR improve outcomes? A single-center experience. Radiat. Oncol. J..

[26] Ost P., Jereczek-Fossa B.A., As N.V., Zilli T., Muacevic A., Olivier K., Henderson D., Casamassima F., Orecchia R., Surgo A. (2016). Progression-free Survival Following Stereotactic Body Radiotherapy for Oligometastatic Prostate Cancer Treatment-naive Recurrence: A Multi-institutional Analysis. Eur. Urol..

[27] Vogelius I.R., Bentzen S.M. (2013). Meta-analysis of the alpha/beta ratio for prostate cancer in the presence of an overall time factor: Bad news, good news, or no news?. Int. J. Radiat. Oncol. Biol. Phys..

[28] Cui M., Gao X.S., Li X., Ma M., Qi X., Shibamoto Y. (2022). Variability of alpha/beta ratios for prostate cancer with the fractionation schedule: Caution against using the linear-quadratic model for hypofractionated radiotherapy. Radiat. Oncol..

[29] Park Y., Park H.J., Jang W.I., Jeong B.K., Kim H.J., Chang A.R. (2018). Long-term results and PSA kinetics after robotic SBRT for prostate cancer: Multicenter retrospective study in Korea (Korean radiation oncology group study 15-01). Radiat. Oncol..

[30] Alongi P., Laudicella R., Lanzafame H., Farolfi A., Mapelli P., Picchio M., Burger I.A., Iagaru A., Minutoli F., Evangelista L. (2022). PSMA and Choline PET for the Assessment of Response to Therapy and Survival Outcomes in Prostate Cancer Patients: A Systematic Review from the Literature. Cancers.

