# Supplementary material for: Radiation Oncologists’ Perspectives on Oligometastatic Prostate Cancer: A Survey from Korean Oligometastasis Working Group
Source: Curr Oncol. 2024 Jun 3;31(6):3239–51. doi: 10.3390/curroncol31060245 (PMC11203304; doi:10.3390/curroncol31060245)
Supplement: Supplementary file 1 [file curroncol-31-00245-s001.zip › curroncol-3019397-supplementary.pdf]

Supplementary Table S1. Questions and corresponding responses for Case 1.

Questions	Options	Total responder	Average # of new patients treated by responders per month			Average # of OM patients treated by responders per year		
			< 30	≥ 30	*P* value	< 10	≥ 10	*P* value
**Q1. Does this case correspond with OMPC?**	**A. Yes**	**93.0% (40/43)**	95.8% (23/24)	89.5% (17/19)	0.575	95.5% (21/22)	90.5% (19/21)	0.607
	B. No	7.0% (3/43)	4.2% (1/24)	10.5% (2/19)		4.5% (1/22)	9.5% (2/21)	
Q2. Which diagnostic modality is required additionally to confirm the OMPC status? (multiple choice)	A. Not required	27.9% (12/43)	25.0% (6/24)	31.6% (6/19)	0.738	18.2% (4/22)	38.1% (8/21)	0.185
	B. PSMA-PET	60.5% (26/43)	12.0% (12/24)	73.7% (14/19)	0.133	54.5% (12/22)	66.7% (14/21)	0.537
	C. Choline PET	0.0% (0/43)	0.0% (0/24)	0.0% (0/19)	NA	0.0% (0/22)	0.0% (0/21)	NA
	D. F-18 FDG PET	32.6% (14/43)	41.7% (10/24)	21.1% (4/19)	0.199	40.9% (9/22)	23.8% (5/21)	0.332
	E. whole spine MRI	20.9% (9/43)	33.3% (8/24)	5.3% (1/19)	0.055	22.7% (5/22)	19.0% (4/21)	1.000
**Q3. Which scheme of systemic therapy do you recommend in this case? (multiple choice)**	**A. ADT for 18 to 36 months**	**79.1% (34/43)**	79.2% (19/24)	78.9% (15/19)	1.000	77.3% (17/22)	81.0% (17/21)	1.000
	B. ADT but if local treatment is applied and PSA levels are	27.9% (12/43)	33.3% (8/24)	21.1% (4/19)	0.500	31.8% (7/22)	23.8% (5/21)	0.736

	undetectable, discontinuation of ADT if there is no evidence of BCR							
	C. Systemic therapy is not recommended.	2.3% (1/43)	0.0% (0/24)	5.3% (1/19)	0.442	0.0% (0/22)	4.8% (1/21)	0.488
**Q4. Which local treatment do you recommend for primary tumor? (multiple choice)**	A. Definitive treatment is not recommended.	7.0% (3/43)	8.3% (2/24)	5.3% (1/19)	1.000	4.5% (1/22)	9.5% (2/21)	0.607
	B. RP + PLND	0.0% (0/43)	0.0% (0/24)	0.0% (0/19)	NA	0.0% (0/22)	0.0 (0/21)	NA
	C. RP + PLND + adjuvant RT	14.0% (6/43)	12.5% (3/24)	15.8% (3/19)	1.000	13.6% (3/22)	14.3% (3/21)	1.000
	**D. Definitive RT**	**93.0% (40/43)**	91.7% (22/24)	94.7% (18/19)	1.000	90.9% (20/22)	95.2% (20/21)	1.000
**Q5. What local therapy do you recommend for bone metastasis in this case? (multiple choice)**	A. Local treatment is not recommended.	4.7% (2/43)	8.3% (2/24)	0.0% (0/19)	0.495	0.0% (0/22)	9.5% (2/21)	0.233
	B. Surgical resection	0.0% (0/43)	0.0% (0/24)	0.0% (0/19)	NA	0.0% (0/22)	0.0% (0/21)	NA
	**C. RT**	**97.7% (42/43)**	95.8% (23/24)	100.0% (19/19)	1.000	100.0% (22/22)	95.2% (20/21)	0.488
	D. Other local therapy, such as local ablative therapy	2.3% (1/43)	4.2% (1/24)	0.0% (0/19)	1.000	4.5% (1/22)	0.0% (0/21)	1.000
**Q6. If you recommend local therapy for bone**	**A. Concurrently with local treatment for primary lesion**	**93.0% (40/43)**	87.5% (21/24)	100.0% (19/19)	0.243	95.5% (21/22)	90.5% (19/21)	0.607

**metastasis, when do you think it is the right time to administer local therapy? (multiple choice)**	B. When symptoms develop during follow-up with other treatments	16.3% (7/43)	25.0% (6/24)	5.3% (1/19)	0.112	13.6% (3/22)	19.0% (4/21)	0.698
	C. When PSA increases during follow-up with other treatments	11.6% (5/43)	3.0% (3/24)	10.5% (2/19)	1.000	13.6% (3/22)	9.5% (2/21)	1.000
	D. When progression is shown on imaging studies during follow-up with other treatments	11.6% (5/43)	3.0% (3/24)	10.5% (2/19)	1.000	9.1% (2/22)	14.3% (3/21)	0.664
Q7. If you recommend RT for bone metastasis, which dose-fractionation regimen do you recommend? (multiple choice)	A. Palliative RT dose for bone metastasis (eg. 30 Gy/10–12 fx, 20 Gy/4–5fx, 8 Gy/1fx, etc. or similar dose-fx scheme)	16.3% (7/43)	25.0% (6/24)	5.3% (1/19)	0.112	18.2% (4/22)	14.3% (3/21)	1.000
	B. SBRT (≤ 5 fractions) (eg. 18–24 Gy/1 fx, 20–24 Gy/2 fx, 21–30 Gy/3 fx, etc. or similar dose-fx scheme)	58.1% (25/43)	50.0% (12/24)	68.4% (13/19)	0.351	45.5% (10/22)	71.4% (15/21)	0.124
	C. Moderate dose RT (≥ 6 fractions) (eg. 30–36 Gy/6 fx, 40–48 Gy/8 fx, 40–50 Gy/10 fx, 39–45Gy/13–15 fx, etc. or similar dose-fx scheme)	41.9% (18/43)	50.0% (12/24)	31.6% (6/19)	0.351	59.1% (13/22)	23.8% (5/21)	**0.031**
	D. Definitive dose with conventional fx (eg. 50–60 Gy in 20–30 fx, etc. with 1.8–2.0 Gy per fx)	34.9% (15/43)	33.3% (8/24)	36.8% (7/19)	1.000	50.0% (11/22)	19.0% (4/21)	0.055
**Q8. When curative local therapy is applied to**	**A. Progression of treated lesion on imaging studies**	**76.7% (33/43)**	70.8% (17/24)	84.2% (16/19)	0.470	86.4% (19/22)	66.7% (14/21)	0.162

**primary and metastatic lesions, which cases do you consider a treatment failure? (multiple choices)**	**B. Newly developed lesion**	**74.4% (32/43)**	70.8% (17/24)	78.9% (15/19)	0.728	72.7% (16/22)	76.2% (16/21)	1.000
	**C. Elevation of PSA**	**86.1% (37/43)**	83.3% (20/24)	89.5% (17/19)	0.678	81.8% (18/22)	90.5% (19/21)	0.664
	D. Not candidate for curative local therapy	2.3% (1/43)	4.2% (1/24)	0.0% (0/19)	1.000	0.0% (0/22)	4.8% (1/21)	0.488

number, *OM* oligometastasis, *OMPC* oligometastatic prostate cancer, *PSMA* prostate specific membrane antigen, *PET* positron-emission tomography, *F-18 FDG* F-18 fluorodeoxyglucose, *MRI* magnetic resonance imaging, *ADT* androgen-deprivation therapy, *BCR* biochemical recurrence, *RP* radical prostatectomy, *PLND* pelvic lymph node dissection, *RT* radiation therapy, *PSA* prostate-specific antigen, *fx* fraction, *SBRT* stereotactic body radiation therapy.

Supplementary Table S2. Questions and corresponding responses for Case 2.

Questions	Options	Total responder	Average # of new patients treated by responders per month			Average # of OM patients treated by responders per year		
			< 30	≥ 30	*P* value	< 10	≥ 10	*P* value
Q1. Does this case correspond with OMPC?	A. Yes	69.8% (30/43)	70.8% (17/24)	68.4% (13/19)	1.000	68.2% (15/22)	71.4% (15/21)	1.000
	B. No	30.2% (13/43)	29.2% (7/24)	31.6% (6/19)		31.8% (7/22)	28.6% (6/21)	
Q2. Could a sudden surge of PSA be a reason for ruling out the possibility of OMPC?	A. Yes	30.2% (13/43)	25.0% (6/24)	36.8% (7/19)	0.509	27.3% (6/22)	33.3% (7/21)	0.747
	B. No	69.8% (30/43)	75.0% (18/24)	63.2% (12/19)		72.7% (16/22)	66.7% (14/21)	
Q3. Which local therapy do you recommend for metastatic lesions in this case? (multiple choice)	A. Not local therapy but systemic therapy or surveillance is recommended (local therapy is only considered as palliative therapy for when related symptom is present).	44.2% (19/43)	50.0% (12/24)	36.8% (7/19)	0.538	50.0% (11/22)	38.1% (8/21)	0.543
	B. RT for LN, lung, and bone metastatic lesions	58.1% (25/43)	54.2% (13/24)	63.2% (12/19)	0.756	45.5% (10/22)	71.4% (15/21)	0.124
	C. Surgical resection for LN and lung metastases, and RT for bone metastases	2.3% (1/43)	4.2% (1/24)	0.0% (0/19)	1.000	0.0% (0/22)	4.8% (1/21)	0.488

	D. Surgical resection for lung metastases, RT for LN and bone metastases	44.2% (19/43)	45.8% (11/24)	42.1% (8/19)	1.000	36.4% (8/22)	50.0 (11/22)	0.364
Q4. If you recommend local therapy for metastatic lesions, when do you think it is the right time to administer local therapy? (multiple choice)	A. Concurrently with systemic therapy	39.5% (17/43)	37.5% (9/24)	42.1% (8/19)	1.000	40.9% (9/22)	38.1% (8/21)	1.000
	B. Prior to systemic therapy	2.3% (1/43)	4.2% (1/24)	0.0% (0/19)	1.000	4.5% (1/22)	0.0% (0/21)	1.000
	C. 2-3 months after initiation of systemic therapy	51.2% (22/43)	45.8% (11/24)	57.9% (11/19)	0.543	45.5% (10/22)	57.1% (12/21)	0.547
	D. When lesion-related symptoms develop while maintaining systemic therapy	25.6% (11/43)	41.7% (10/24)	5.3% (1/19)	**0.012**	27.3% (6/22)	23.8% (5/21)	1.000
	E. When PSA rises during maintenance of systemic therapy	25.6% (11/43)	20.8% (5/24)	31.6% (6/19)	0.495	22.7% (5/22)	28.6% (6/21)	0.736
Q5. If you recommend RT for bone metastasis, what do you think of the appropriate dose? (multiple choice)	A. Palliative RT dose for bone metastasis (eg. 30 Gy/10-12 fx, 20 Gy/4-5fx, 8 Gy/1fx, etc. or similar dose-fx scheme)	23.3% (10/43)	37.5% (9/24)	5.3% (1/19)	**0.026**	27.3% (6/22)	19.0% (4/21)	0.721
	B. SBRT (≤ 5 fractions) (eg. 18-24 Gy/1 fx, 20-24 Gy/2 fx, 21-30 Gy/3 fx, etc. or similar dose-fractionation scheme)	69.8% (30/43)	62.5% (15/24)	78.9% (15/19)	0.324	59.1% (13/22)	81.0% (17/21)	0.185

	C. Moderate dose RT (≥ 6 fractions) (eg. 30–36 Gy/6 fx, 40–48 Gy/8 fx, 40–50 Gy/10 fx, 39-45Gy/13–15 fx, etc. or similar dose-fx scheme)	30.2% (13/43)	29.2% (7/24)	31.6% (6/19)	1.000	31.8% (7/22)	28.6% (6/21)	1.000
	D. Definitive dose with conventional fx (eg. 50–60 Gy in 20–30 fx, etc. with 1.8–2.0 Gy per fx)	4.7% (2/43)	4.2% (1/24)	5.3% (1/19)	1.000	9.1% (2/22)	0.0% (0/21)	0.488
Q6 If you recommend RT for LN metastasis, which dose-fractionation regimen and field do you recommend? (multiple choice)	A. Palliative RT to LN metastasis only	7.0% (3/43)	12.5% (3/24)	0.0% (0/19)	0.243	13.6% (3/22)	0.0% (0/21)	0.233
	B. Definitive hypofractionated RT to LN metastasis only	67.4% (29/43)	70.8% (17/24)	63.2% (12/19)	0.745	59.1% (13/22)	76.2% (16/21)	0.332
	C. Definitive conventional fractionated RT to LN metastasis only	14.0% (6/43)	12.5% (3/24)	15.8% (3/19)	1.000	9.1% (2/22)	19.0% (4/21)	0.412
	D. Definitive conventional fractionated RT to whole pelvis	23.3% (10/43)	20.8% (5/24)	26.3% (5/19)	0.728	27.3% (6/22)	19.0% (4/21)	0.721
Q7. If you recommend RT for pelvic LN metastasis, is it necessary to include a prostate bed in the RT field?	A. Yes	32.6% (14/43)	33.3% (8/24)	31.6% (6/19)	1.000	40.9% (9/22)	23.8% (5/21)	0.332
	B. No	67.4% (29/43)	66.7% (16/24)	68.4% (13/19)		59.1% (13/22)	76.2% (16/21)	
Q8. If you recommend RT for lung metastases, which dose-fractionation	A. Palliative RT dose (eg. 30 Gy/10–12 fx, 20 Gy/4–5fx, 8 Gy/1fx, etc. or similar dose-fx cheme)	9.3% (4/43)	12.5% (3/24)	5.3% (1/19)	0.618	13.6% (3/22)	4.8% (1/21)	0.607

regimen do you recommend? (multiple Choice)	B. SBRT (≤ 5 fx with BED_{10} ≥ 100 Gy	62.8% (27/43)	54.2% (13/24)	73.7% (14/19)	0.221	45.5% (10/22)	81.0% (17/21)	**0.027**
	C. SBRT (≤ 5 fractions) with BED_3 ≥ 100 Gy	32.6% (14/43)	25.0% (6/24)	42.1% (8/19)	0.329	40.9% (9/22)	23.8% (5/21)	0.332
	D. Moderate dose RT (more than 6 fractions) (eg. 30–36 Gy/6 fx, 40–48 Gy/8 fx, 40–50 Gy/10 fx, 39–45Gy/13–15 fx, etc. or similar dose-fractionation scheme)	34.9% (15/43)	33.3% (8/24)	36.8% (7/19)	1.000	40.9% (9/22)	28.6% (6/21)	0.526
	E. Definitive dose with conventional fx (eg. 50–60 Gy in 20–30 fx, etc. with 1.8–2.0 Gy per fx)	7.0% (3/43)	8.3% (2/24)	5.3% (1/19)	1.000	13.6% (3/22)	0.0% (0/21)	0.233
**Q9. If curative local therapy is applied to metastatic lesions, when do you consider it as a treatment failure? (multiple choice)**	**A. When the progression of treated lesion seen on imaging studies**	**81.4% (35/43)**	79.2% (19/24)	84.2% (16/19)	1.000	81.8% (18/22)	81.0% (17/21)	1.000

	**B. When new lesion(s) developed**	**72.1% (31/43)**	66.7% (16/24)	78.9% (15/19)	0.500	72.7% (16/22)	71.4% (15/21)	1.000
	**C. When the level of PSA elevated**	**72.1% (31/43)**	75.0% (18/24)	68.4% (13/19)	0.738	72.7% (16/22)	71.4% (15/21)	1.000

OMPC oligometastatic prostate cancer, *PSA* prostate-specific antigen, *RT* radiation therapy, *LN* lymph node, *fx* fraction, *SBRT* stereotactic body radiation therapy; BED_x biological effective dose with $\alpha/\beta=x$.

Supplementary Table S3. Questions and corresponding responses for Case 3.

Questions	Options	Total responder	Average # of new patients treated by responders per month			Average # of OM patients treated by responders per year		
			< 30	≥ 30	*P* value	< 10	≥ 10	*P* value
Q1. Does this case correspond with OMPC?	A. Yes	46.2% (18/39)	36.4% (8/22)	58.8% (10/17)	0.206	36.8% (7/19)	55.0% (11/20)	0.341
	B. No	53.8% (21/39)	63.6% (14/22)	41.2% (7/17)		63.2% (12/19)	45.0% (9/20)	
Q2. If this case is not included in the category of OMPC, what can be the reason? (multiple choice)	A. History of polymetastasis	53.8% (21/39)	68.2% (15/22)	35.3% (6/17)	0.057	63.2% (12/19)	45.0% (9/20)	0.341
	B. Progression to CRPC	15.4% (6/39)	18.2% (4/22)	11.8% (2/17)	0.679	26.3% (5/19)	5.0% (1/20)	0.091
	C. Insufficiency in test for confirmation of OMPC	2.6% (1/39)	4.5% (1/22)	0.0% (0/17)	1.000	5.3% (1/19)	0.0% (0/20)	0.487
	D. This case is included in the category of OMPC.	46.2% (18/39)	31.8% (7/22)	64.7% (11/17)	0.057	36.8% (7/19)	55.0% (11/20)	0.341
**Q3. Do you recommend local therapy (RT or surgery, etc.) for all metastatic lesions in this case?**	**A. Yes**	**71.8% (28/39)**	63.6% (14/22)	82.4% (14/17)	0.288	63.2% (12/19)	80.0% (16/20)	0.301
	B. No (local therapy should be considered when lesion-related symptoms occur.)	28.2% (11/39)	36.4% (8/22)	17.6% (3/17)		36.8% (7/19)	20.0% (4/20)	
Q4. If you recommend RT for metastatic lesions,	A. Palliative RT dose (eg, 30 Gy/10–12 fx, 20 Gy/4–5fx, 8	17.9% (7/39)	27.3% (6/22)	5.9% (1/17)	0.113	26.3% (5/19)	10.0% (2/20)	0.235

which dose-fractionation regimen do you recommend? (multiple choice)	Gy/1fx, etc. or similar dose-fx scheme)							
	B. SBRT (≤ 5 fx) with $BED_{10} \geq 100$ Gy	61.5% (24/39)	54.5% (12/22)	70.6% (12/17)	0.343	42.1% (8/19)	80.0% (16/20)	**0.022**
	C. SBRT (≤ 5 fx) with $BED_3 \geq 100$ Gy	23.1% (9/39)	13.6% (3/22)	35.3% (6/17)	0.142	31.6% (6/19)	15.0% (3/20)	0.273
	D. Moderate dose RT (more than 6 fx) (eg. 30–36 Gy/6 fx, 40–48 Gy/8 fx, 40–50 Gy/10 fx, 39–45Gy/13–15 fx, etc. or similar dose-fx scheme)	35.9% (14/39)	31.8% (7/22)	41.2% (7/17)	0.738	31.6% (6/19)	40.0% (8/20)	0.741
	E. Definitive dose with conventional fx (eg. 50–60 Gy in 20–30 fx, etc. with 1.8–2.0 Gy per fx)	2.6% (1/39)	0.0% (0/22)	5.9% (1/17)	0.436	5.3% (1/19)	0.0% (0/20)	0.487
Q5. If RT for metastatic lesions is recommended, do you think PSMA-PET is necessary to evaluate remission after RT?	A. Yes	61.5% (24/39)	68.2% (15/22)	52.9% (9/17)	0.508	68.4% (13/19)	55.0% (11/20)	0.514
	B. No	38.5% (15/39)	31.8% (7/22)	47.1% (8/17)		31.6% (6/19)	45.0% (9/20)	

**Q6. If you have achieved local control of metastatic lesions after local therapy, do you think it is necessary to maintain systemic therapy?**	**A. Yes**	**87.2% (34/39)**	90.9% (20/22)	82.4% (14/17)	0.636	84.2% (16/19)	90.0% (18/20)	0.661
	B. No	12.8% (5/39)	9.1% (2/22)	17.6% (3/17)		15.8% (3/19)	10.0% (2/20)	

OMPC oligometastatic prostate cancer, *CRPC* castration-resistant prostate cancer, *RT* radiation therapy, *fx* fraction(s), *SBRT* stereotactic body radiation therapy, BED_x biological effective dose with α/β=x, *PSMA-PET* prostate specific membrane antigen positron-emission tomography.
